# The Impact of Transcatheter or Surgical Defect Closure on Self-Reported Sleep Quality in Adults with Atrial Septal Defect

**DOI:** 10.21470/1678-9741-2023-0267

**Published:** 2024-04-03

**Authors:** Mert Evlice, Sinem Berik Safçı, Emre Paçacı, Samet Ayna, Sinan Cerşit, Lütfi Öcal, Mustafa Ozan Gürsoy, Abdullah Yıldırım, İbrahim Halil Kurt

**Affiliations:** 1 Department of Cardiology, Health Sciences University, Adana City Training and Research Hospital, Adana, Turkey; 2 Department of Pulmonology, Health Sciences University, Adana City Training and Research Hospital, Adana, Turkey; 3 Department of Cardiology, Health Sciences University, Kartal Koşuyolu Training and Research Hospital, İstanbul, Turkey; 4 Department of Cardiology, Health Sciences University, İzmir Bozyaka Training and Research Hospital, İzmir, Turkey

**Keywords:** Atrial Septal Defect, Pittsburgh Sleep Quality Index, Sleep Quality, Surgical Defect Closure, Transcatheter Defect Closure

## Abstract

**Objective:**

Sleep quality in those with cardiovascular disease is significantly lower than in the general population. This study aimed to explore the effect of transcatheter or surgical closure of atrial septal defect (ASD) on sleep quality.

**Methods:**

One hundred nineteen adult patients with ASD who underwent transcatheter or surgical closure were included in the study. Sleep quality was investigated prospectively just before defect closure and six months after defect closure. Pittsburgh Sleep Quality Index (PSQI) was used to evaluate sleep quality of these patients.

**Results:**

PSQI scores were similar in both groups before the procedure in patients who underwent both transcatheter and surgical closure. The PSQI scores six months after transcatheter closure was significantly improved compared to the PSQI score before transcatheter ASD closure (3.5 ± 2.0 *vs.* 6.9 ± 3.4, respectively; *P*<0.001). The PSQI scores six months after surgical ASD closure was significantly improved compared to the PSQI score before surgical closure (4.8 ± 2.1 *vs.* 7.1 ± 2.0, respectively; *P*<0.001). Total PSQI scores were also statistically different at six months after transcatheter and surgical closure (3.5 ± 2.0 *vs.* 4.8 ± 2.1, *P*=0.014). However, six months after both transcatheter and surgical closure, PSQI scores were significantly decreased in both groups which was more pronounced in patients who underwent transcatheter closure.

**Conclusion:**

Transcatheter or surgical closure of the defect may be beneficial in improving the sleep quality of adult patients with ASD. Delayed improvement of sleep quality after surgical closure may be an important advantage for transcatheter closure.

## INTRODUCTION

Atrial septal defect (ASD) represents the most common congenital heart disease (CHD) diagnosed in adulthood. ASD accounts for 25-30% of newly diagnosed CHDs in adulthood. There are four main types of ASD: ostium primum, ostium secundum, sinus venosus, and unroofed type ASD. The most common form of ASD is ostium secundum^[1,2]^. In adult patients with ASD, a left-to-right shunt is responsible for right ventricular volume overload and increased pulmonary circulation. Defects with a diameter ≤ 10 mm are responsible for some degree of shunting (pulmonary-systemic shunt ratio [Qp/Qs] < 1.5). However, these defects usually do not cause right ventricular volume overload. Larger diameter defects (> 10 mm) may cause volume overload in the right heart chambers. Therefore, these defects cause enlargement of the right heart chambers, lead to an increase in central venous pressure, and development of pulmonary arterial hypertension^[1]^.

Although ASD is considered a simple defect in CHDs, it is associated with significant late-term morbidity and mortality if not treated appropriately^[2]^. In adult patients with ASD with evidence of right ventricular overload, transcatheter or surgical closure of the ASD is recommended, provided there is no significant pulmonary arterial hypertension^[2,3]^. Thanks to advances in medical imaging, many simple ASDs are diagnosed and treated in adulthood. Due to the advancement of medical equipment and surgical technology, transcatheter intervention is widely used in clinical practice for simple ASD and even some complex ASD^[4,5]^.

Sleep is a cyclical, temporary, and functional state in which an average of one-third of human life is spent, primarily controlled by neurobiological processes^[6]^. Recent studies have shown that sleep deprivation or reduced quality has a strong effect on the occurrence and prognosis of many important diseases, including cardiovascular diseases, cancer, depression, obesity, and immune system dysfunction^[7]^.

Good sleep quality is one of the most important factors contributing to physical functionality, psychological well-being, and quality of life. Good sleep may also play a protective role in terms of cardiovascular diseases that may develop later on. Sleep quality is often used to express a set of sleep measures such as total sleep time, sleep onset latency, sleep efficiency, wakefulness after sleep onset, and daytime sleepiness^[8,9]^. Sleep quality is affected by many variables such as diet, physical activity, genetics, environmental factors, and comorbidities^[6]^. Polysomnography (PSG) is the gold standard method for assessing sleep duration, quality, and architecture. However, due to its cost and limited availability, questionnaires to evaluate sleep quality are usually applied in large populations^[10]^.

In current literature, there is lacking data regarding sleep quality of patients with ASD and sleep quality after transcatheter and surgical ASD closure. The aim of this study was to evaluate the patients’ self-assessed responses to sleep quality six months after transcatheter and surgical ASD closure, and to examine echocardiographic variables that may also be associated with sleep quality in patients with ASD.

## METHODS

### Study Design

One hundred nineteen adult patients with ASD who underwent successful transcatheter or surgical ASD closure in the Department of Cardiology of Adana City Training and Research Hospital between January 2021 and March 2023 were included in the study. All patients included in the study underwent transesophageal echocardiography after the initial transthoracic echocardiographic study. In addition, cardiac catheterization was performed in selected patients. It was evaluated by the heart team, which included the cardiologist, cardiovascular surgeon, and anesthesiologist. The heart team made a decision between surgical and transcatheter ASD closure. For the indication of ASD closure, the recommendations of the adult CHD guideline of the European Society of Cardiology published in 2020 were taken into consideration. According to this guideline^[5]^, all patients with increased pulmonary flow (Qp/Qs > 1.5) and pulmonary vascular resistance (PVR) (< 5 woods) underwent transcatheter or surgical intervention for the treatment of ASD.

Pittsburgh Sleep Quality Index (PSQI) questionnaire was administered to all patients who were planned for surgery or transcatheter closure due to ASD, before the procedure and at six months after the procedure.

Patients with ASD with small (defect diameter < 10 mm, Qp/Qs < 1.5) defects, patients with ASD and severe pulmonary hypertension (PVR > 5 woods), patients with additional cardiac defects to ASD, and patients whose ASD was a component of complex CHD were not included in this study. Furthermore, patients with significant left heart diseases (rheumatic heart diseases, cardiomyopathies, coronary artery disease, and heart failure), cardiac arrhythmias (including use of antiarrhythmic drugs), significant lung pathologies (asthma, sleep apnea syndrome, and chronic obstructive pulmonary disease), and chest deformities (pectus excavatum, pectus carinatum) were not included in the study. Patients with active thyroid disease, chronic anemia, malignancy, pregnancy (including suspected pregnancy), chronic kidney disease, known psychiatric disorders, active infectious disease, patients receiving treatment affecting sleep quality, and those who refused to participate in the survey were also excluded from the study.

Sleep quality was investigated prospectively just before defect closure and six months after defect closure. PSQI was used to evaluate the sleep quality of these patients. The quantitative component of the study collected data from participants to determine self-rated sleep quality through completing a series of scales in this questionnaire. The subjects who might have impaired sleep quality before and after the procedure due to depression, anxiety, another concomitant disease, and those who used a sedative drug for pain were excluded. This questionnaire was applied to all patients participating in the study only twice, before defect closure and six months after defect closure. A total of 238 surveys were conducted, and all of them were actually recovered, resulting in a 100% recovery rate. After checking the validity and completeness of the questionnaire, it was found that the effectiveness and completeness of the questionnaire were 100%. PSQI is a questionnaire compiled mainly to evaluate the sleep quality of patients with sleep disorders and mental disorders^[8,9]^. Moreover, it is a suitable questionnaire for assessing the sleep quality of ordinary people. The survey consists of nine questions in total. The first four questions are fill-in-the-blank questions. The last five are multiple-choice questions. Also, the fifth question contains 10 small questions. The 18 self-assessment items consist of seven components, which are: 1. subjective sleep quality; 2. sleep latency (delay); 3. sleep duration; 4. habitual sleep efficiency; 5. sleep disturbance; 6. sleep medication use; and 7. daytime dysfunction. Each of these components is scored on a scale from 0 to 3. The cumulative score of each component is the total PSQI score, and the total score ranges from 0 to 21 points. A high total score indicates poor sleep quality. The lower the score, the better the sleep quality^[8,9]^.

### Ethical Considerations

The Institutional Review Board of our Hospital approved this study (approval number: 12-2021-2346). Written informed consent to participate in the study was obtained from all participants. The principles of the study are in accordance with the Declaration of Helsinki.

### Statistical Analysis

Pre-intervention and six-month post-intervention analyses of scores for all aspects of sleep quality were used to test the hypothesis that ASD closure could improve sleep quality. All data were numerically encoded. For statistical analysis, they were entered into the IBM Corp. Released 2013, IBM SPSS Statistics for Windows, version 22.0, Armonk, NY: IBM Corp. computer software package and scanned for variable and case-by-case missing values. Quantitative data were expressed as mean ± standard deviation. Qualitative data were compared between groups using the chi-square test. Paired *t*-test was used to analyze the sleep quality data before and six months after ASD closure. Correlation analysis was performed between total PSQI scores to compare sleep quality before and six months after ASD closure.

## RESULTS

One hundred nineteen patients with ASD were included in the study. Of these patients, eight had ostium primum type ASD, 94 had ostium secundum type ASD, 16 had superior sinus venosus type ASD, and one had inferior sinus venosus type ASD. Of 16 patients with superior sinus venosus type ASD, three had partial pulmonary venous return anomaly and four had persistent left superior vena cava. Of 94 patients with ostium secundum type ASD, four had partial pulmonary venous return anomaly and two had persistent left superior vena cava.

Of the 119 patients who participated in the study, 76 were female (63.9%) and 43 were male (36.1%), with a mean age of 39.9 ± 10 years. The baseline characteristics such as age, sex, and weight of all patients are documented in Table 1. The patients were divided into two groups as transcatheter (n=84) and surgical (n=35) ASD closure groups. Before transcatheter and surgical ASD closure, there was no statistically significant difference between the two groups in terms of many demographic, clinical, and laboratory parameters (Table 1). However, the surgical ASD closure group consisted of subjects with larger ostium secundum type ASD unsuitable for transcatheter closure, and sinus venous and ostium primum ASD cases who had no indication for transcatheter closure.

**Table 1:** Comparison of echocardiographic data before defect closure in transcatheter and surgical ASD closure groups.

Parameters	Transcatheter closure group (n: 84)	Surgical closure group (n: 35)	*P*-value
Age, years	39.2 ± 10.1	41.4 ± 9.8	0.28
Sex, male, n (%)	31 (37%)	12 (34%)	0.79
Smoking, n (%)	31 (37%)	12 (34%)	0.79
BMI, kg/m^2^	27.1 ± 4.1	27.7 ± 3.8	0.42
Heart rate, bpm	82.9 ± 14.5	81.1 ± 11.1	0.5
Systolic blood pressure, mmHg	118.5 ± 17.6	118.9 ± 14.6	0.9
Diastolic blood pressure, mmHg	72.7 ± 11.9	72.8 ± 10.6	0.95
C-reactive protein, mg/L	5.2 ± 1.5	4.9 ± 1.3	0.74
Thyroid-stimulating hormone, mUI/L	4.1 ± 1.3	3.9 ± 1.6	0.78
Hemoglobin, gr/dL	13.1 ± 2.8	13.3 ± 2.7	0.71
Albumin, gr/dL	42 ± 3.7	39.8 ± 3.5	0.93
Total bilirubin, mg/dL	0.64 ± 0.44	0.65 ± 0.33	0.92
Alanine aminotransferase, U/L	16.8 ± 7.4	18.2 ± 8.6	0.39
Aspartate aminotransferase, U/L	23.2 ± 7.3	22.8 ± 7.2	0.75
NT-proBNP, µg/L	246.2 ± 150.5	257.9 ± 162.1	0.74
Total PSQI (before defect closure)	6.9 ± 3.4	7.1 ± 2.5	0.9
Total PSQI (after defect closure)	3.5 ± 2.0	4.8 ± 2.1	0.014*

ASD=atrial septal defect; BMI=body mass index; NT-proBNP=N-terminal pro brain natriuretic peptide; PSQI=Pittsburgh Sleep Quality Index

*The difference was statistically significant (*P*<0.05)

There was no statistically significant difference in total PSQI scores of the patients who were planned for transcatheter and surgical ASD closure (6.9 ± 3.4 *vs.* 7.1 ± 2.5, *P*=0.9). Total PSQI scores were statistically different at six months after transcatheter and surgical ASD closure (3.5 ± 2.0 *vs.* 4.8 ± 2.1, *P*=0.014*). The total PSQI score was better in favor of transcatheter ASD closure. Transcatheter or surgical ASD closure was compared before and after six months. There was an improvement in the PSQI score, a subjective measure of sleep quality. In patients who underwent transcatheter closure due to ASD, PSQI was significantly improved and correlated before and at six months after the procedure (2.5 ± 2.2 scores, t=10.5, *P*<0.001) (r=0.8, *P*<0.001). Similarly, in patients who underwent surgical closure due to ASD, PSQI was significantly improved and correlated before the procedure and at six months after the procedure (1.3 ± 1.5 scores, t=5.3, *P*<0.001) (r=0.7, *P*<0.001). However, this improvement was better in the transcatheter ASD closure group compared to the surgical ASD closure group.

There were no significant differences in echocardiographic parameters between the two groups before defect closure (Table 2). A significant univariate correlation was demonstrated between total PSQI scores and echocardiographic parameters (right ventricular diameter [r=0.505, *P*=0.001*], right atrial diameter [r=0.420, *P*=0.001*], estimated systolic pulmonary artery pressure [r=0.464, *P*=0.001*], Qp/Qs [r=0.660, *P*=0.001*]) (Table 3). In addition, multivariate regression analysis was performed between correlated echocardiographic parameters and total PSQI scores. Right ventricular diameter (β=0.210, *P*=0.015**) and Qp/Qs (β=0.514, *P*=0.001*) were considered statistically significant (Table 3).

**Table 2:** Comparison of echocardiographic data before defect closure in transcatheter and surgical ASD closure groups.

Parameters	Transcatheter closure group	Surgical closure group	*P*-value
Left ventricular EF, %	66.4 ± 3.1	65.9 ± 3.2	0.41
Left atrial diameter, mm	33.3 ± 4.7	34.2 ± 4.2	0.31
Right ventricular diameter, mm	37.5 ± 4.7	39 ± 4.4	0.12
Right atrial diameter, mm	41.7 ± 5.4	42.6 ± 5.8	0.40
Estimated PAP, mmHg	36.3 ± 9.4	36.7 ± 11	0.87
Qp/Qs	2.3 ± 0.7	2.6 ± 0.6	0.31
TAPSE, mm	22.3 ± 3.4	22.1 ± 3.1	0.78

ASD=atrial septal defect; EF=ejection fraction; PAP=pulmonary artery pressure; Qp/Qs=pulmonary-systemic shunt ratio; TAPSE=tricuspid annular plane systolic excursion

**Table 3:** The relationship between total PSQI scores and echocardiographic parameters before defect closure in patients with ASD.

	Univariate analysis		Multivariate analysis	
	r-value	*P*-value	β-value	*P*-value
Left ventricular EF	0.073	0.43	-	-
Left atrial diameter	0.098	0.29	-	-
Right ventricular diameter	0.505	0.001*	0.210	0.015*
Right atrial diameter	0.420	0.001*	0.129	0.124
Estimated PAP	0.464	0.001*	0.108	0.2
Qp/Qs	0.660	0.001*	0.514	0.001*
TAPSE	-0.121	0.19	-	-

ASD=atrial septal defect; EF=ejection fraction; PAP=pulmonary artery pressure; PSQI=Pittsburgh Sleep Quality Index. Qp/ Qs=pulmonary-systemic shunt ratio; TAPSE=tricuspid annular plane systolic excursion

*The difference was statistically significant (*P*<0.05)

## DISCUSSION

In this study, while the sleep quality evaluated with the PSQI questionnaire before defect closure was impaired in patients with ASD, a significant improvement in sleep quality was demonstrated at the sixth month after defect closure. The improvement in sleep quality was more prominent in the transcatheter ASD closure group compared to the surgical ASD closure group.

**Fig. 1:**
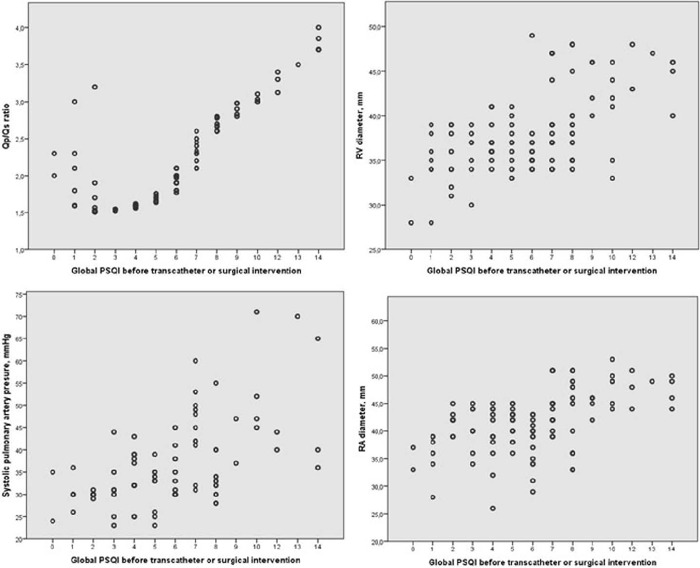
Correlation between echocardiographic parameters and total Pittsburgh Sleep Quality Index (PSQI) before transcatheter or surgical atri-al septal defect closure (pulmonary-systemic shunt ratio [Qp/Qs] [r=0.660, P<0.001*], estimated systolic pulmonary artery pressure [r=0.464, P<0.001*], right ventricular [RV] diameter [r=0.505, P<0.001*], and right atrial [RA] diameter [r=0.420, P<0.001*], respectively). *The difference was statistically significant (P<0.05).

The PSQI score before transcatheter ASD closure was 6.9 ± 3.4, while the PSQI score after transcatheter ASD closure was 3.5 ± 2.0. Sleep quality was considerably higher at six months after transcatheter ASD closure compared to pre-transcatheter ASD closure. The PSQI score before surgical ASD closure was 7.1 ± 2.5, while the PSQI score after surgical ASD closure was 4.8 ± 2.1. The PSQI scores were similar in both groups before the procedure in patients who underwent both transcatheter and surgical ASD closure. However, six months after both transcatheter and surgical ASD closures, PSQI scores were statistically significantly decreased in both groups. This decrease in PSQI score was more pronounced in patients who underwent transcatheter ASD closure (*P*<0.001). Baseline echocardiographic data in patients with ASD were similar between the transcatheter and surgical closure groups (Table 2). When the univariate analysis between total PSQI value and echocardiographic data was examined, a significant correlation was found between total PSQI value and Qp/Qs, estimated systolic PAP, right ventricular diameter, and right atrial diameter (Table 3, Figure 1). In addition, when the multivariate analysis between total PSQI and echocardiographic data was examined, a statistically significant relationship was found between Qp/Qs and right ventricular diameter and total PSQI (Table 3). These data may indicate that an increase in the right heart volume load or the amount of left-right shunt may be associated with deterioration of sleep quality. In addition, due to the increased venous return at night, the right heart volume load will increase slightly, which may have an additional negative effect on sleep quality.

ASD is associated with the development of impaired aerobic capacity corresponding to right heart volume overload, pulmonary arterial hypertension, congestive heart failure, and atrial arrhythmias. Transcatheter or surgical closure of ASD in adulthood is highly effective and safe in reducing or eliminating left-to-right shunts and right heart volume overload. In addition, transcatheter or surgical closure of the ASD prevents the development of pulmonary arterial hypertension and congestive heart failure and provides symptomatic relief^[11,12]^.

Sleep quality is significantly lower in patients with cardiovascular diseases compared to the general population. There are a few studies in the literature about a deterioration in sleep quality in patients with heart failure, coronary artery disease, and cardiac arrhythmia^[13,14,15,16,17]^. However, there are almost no studies in the literature evaluating sleep quality in patients with ASD. ASD represents an important group of congenital cardiac defects in the adult population. It is successfully treated with surgery or transcatheter closure. In our study, we detected deterioration in sleep quality in patients with ASD using the PSQI questionnaire. Moreover, through this questionnaire, we found a statistically significant improvement in sleep quality in both transcatheter and surgical closure groups. A total PSQI score > 5 in the PSQI questionnaire indicates poor sleep quality, that is, sleep quality is impaired^[8,9]^. The PSQI is a subjective measure of nighttime sleep quality with total possible scores ranging from 0 to 21. It is recommended to evaluate PSG as an objective measure of night sleep quality^[10]^. However, its use is limited due to its cost and availability. Since sleep quality could not be evaluated by PSG in our study, the results obtained are subjective, which is an important limitation of the study.

In our study, the improvement in PSQI scores in the surgical ASD closure group was less than in the transcatheter ASD closure group, which may be related to the effect of cardiac surgery-related factors on sleep quality. These factors associated with cardiac surgery may include postoperative pain, postoperative anxiety, postoperative depression, and prolonged duration of postoperative hospital stay. On the other hand, the patients who are treated with transcatheter ASD closure procedure are not exposed to these factors that adversely affect sleep quality. A more significant improvement in subjective sleep quality in patients undergoing transcatheter ASD closure can be considered as a significant advantage when compared to surgical ASD closure.

In a study of patients who had undergone cardiac surgery, the results showed that they continued to have sleep disturbances even six months after discharge from the hospital^[18,19]^.

Redeker et al. found that it takes approximately two months for sleep quality to reach pre-cardiac surgery level, but sleep efficiency remains < 85% even six months after discharge^[20]^. In both studies on sleep after cardiac surgery, the results show that sleep disturbance still persists six months after hospital discharge^[18,19]^. As a result, it shows that sleep quality gradually improves in patients who have had heart surgery. These studies show that complete recovery may not occur even six months after cardiac surgery^[18,19]^. This may explain the reason why PSQI scores improved less in patients who underwent surgical ASD closure compared to patients who underwent transcatheter ASD closure in our study.

More significant improvement in sleep quality in patients undergoing transcatheter ASD closure is an important advantage compared to surgical ASD closure. The patients are not exposed to the factors that adversely affect sleep quality in the transcatheter approach. Because of the long-term and slow improvement in sleep quality after cardiac surgery, less improvement in sleep quality is expected in the surgical ASD closure group compared to the transcatheter approach group.

### Limitations

This is a small-scaled study with a limited follow-up period. It is possible to detect similar efficacy in sleep quality after both interventions during follow-up longer than six months. Therefore, this study should be supported by studies with a larger population and longer follow-up. Furthermore, the factors affecting sleep quality could not be examined in detail in the current study. Future studies combining objective (PSG) and subjective (sleep-related questionnaires) sleep measurements are needed to examine sleep and related factors during the recovery period before or after the procedure in patients with ASD.

## CONCLUSION

Sleep quality may be affected by left-right shunt and right heart volume load, and sleep quality may improve after correction of ASD. Delayed improvement of sleep quality after surgical ASD closure may be an important advantage for transcatheter ASD closure. The study needs to be supported by PSG, which can objectively evaluate sleep quality in a larger ASD population.

## Abbreviations, Acronyms & Symbols

ASD: Atrial septal defect
BMI: Body mass index
CHD: Congenital heart disease
EF: Ejection fraction
NT-proBNP: N-terminal pro brain natriuretic peptide
PAP: Pulmonary artery pressure
PSG: Polysomnography
PSQI: Pittsburgh Sleep Quality Index
PVR: Pulmonary vascular resistance
Qp/Qs: Pulmonary-systemic shunt ratio
RA: Right atrial
RV: Right ventricular
TAPSE: Tricuspid annular plane systolic excursion
